# Learning Curve Analysis of Single-Incision Ovarian Cystectomy: Comparative Study of Robotic and Conventional Laparoscopic Techniques

**DOI:** 10.3390/jpm14080785

**Published:** 2024-07-24

**Authors:** Seongmin Kim, Seon-Mi Lee, Aeran Seol, Sanghoon Lee, Jae-Yun Song, Jae-Kwan Lee, Nak-Woo Lee

**Affiliations:** 1 Gynecologic Cancer Center, CHA Ilsan Medical Center, CHA University College of Medicine, 1205 Jungang-ro, Ilsandong-gu, Goyang-si 10414, Republic of Korea; naiad515@gmail.com; 2 Department of Obstetrics and Gynecology, Korea University College of Medicine, 73 Inchon-ro, Seongbuk-gu, Seoul 02841, Republic of Korea

**Keywords:** ovarian cystectomy, minimally invasive surgery, DaVinci Robotic Single-Site, DaVinci Robotic Single-Port, laparo-endoscopic single-site surgery, learning curve

## Abstract

Ovarian cystectomy, aimed at preserving fertility, has advanced through minimally invasive surgical techniques. This study evaluates the learning curves and surgical outcomes of three such approaches: DaVinci Robotic Single-Site (RSS), DaVinci Robotic Single-Port (RSP), and laparo-endoscopic single-site surgery (LESS). To analyze the learning curves and surgical outcomes for these techniques, providing insights into their effectiveness and proficiency development. Retrospective analysis of 104 patients with ovarian tumors, divided into RSS (*n* = 52), RSP (*n* = 22), and LESS (*n* = 30) groups. Metrics analyzed included age, BMI, tumor size, hemoglobin drop, operative time, docking time, console time, and tumor location. No significant differences in age, BMI, transfusion rate, hemoglobin drop, or length of stay were found among the groups. RSS had larger tumors on average, and LESS had a higher occurrence rate on the right side. LESS demonstrated the shortest operative time, while RSS and RSP had comparable times. Docking and console times did not differ significantly between RSS and RSP. RSP reached proficiency faster than RSS in docking and console times, while LESS exhibited the greatest variability in operative time. RSP offers a faster and more consistent learning curve, making it advantageous for complex procedures, whereas LESS provides shorter operative times but with higher variability. These findings are crucial for surgical training and resource allocation in medical institutions.

## 1. Introduction

Ovarian cystectomy is a common surgical procedure, performed to remove cysts from ovaries [1]. For cases where malignant or borderline ovarian tumors are not suspected, an ovarian cystectomy is performed to remove only the tumor while preserving the ovary, maximizing fertility preservation. Such fertility preservation is often necessary for younger women, who are also more sensitive to surgical scars. Advancements in minimally invasive surgery have introduced various single-incision surgery techniques such as the DaVinci Robotic Single-Site platform (RSS), the DaVinci Robotic Single-Port system (RSP), and laparo-endoscopic single-site surgery (LESS) [2,3].

LESS surgery is increasingly being used in gynecological procedures for young women due to its advantage of minimizing scarring [4]. Furthermore, this technique has been utilized in highly complex surgeries to overcome limitations in angulation and device manipulation. However, surgeons require an extended learning period to attain proficiency and minimize technical difficulties [5]. Among the diverse range of laparoscopic surgical techniques, the intra-corporeal suturing of ovarian tissue after ovarian cystectomy is one of the most difficult procedures to master in a short period.

Robotic surgery technology offers enhanced visualization and surgical precision, enabling surgeons to perform complex laparotomy techniques using minimally invasive approaches. This is largely due to the improved ergonomic design of robotic systems [6]. The DaVinci Single-Site platform is a specialized surgical system designed by Intuitive Surgical (Sunnyvale, CA, USA) for performing single-site surgeries using a DaVinci Si/Xi/X system [7]. The Single-Site platform specifically allows for single-incision surgeries, meaning that the entire procedure can be performed through one small incision, typically in the patient’s navel. One 8 mm umbilical cannula and two 5 mm semi-rigid curved cannulas are used for this platform. Even though RSS surgery may not be as flexible as multiport robotic surgery, it offers advantages in intra-corporeal suturing, preventing instrument collisions, and achieves better triangulation compared to LESS. While there are some limitations associated with the RSS platform, most of these limitations are overcome in the RSP system [8]. The DaVinci RSP surgical system contains four instrument drives that control the articulating camera and up to three robotic instruments that can be simultaneously positioned through a 25 mm SP multichannel port. Unlike previous models of the DaVinci RSS platform, the EndoWrist SP instruments (manufactured by Intuitive Surgical, Sunnyvale, CA, USA) have two joints. The wrist joint allows for 7 degrees of freedom and the elbow joint maintains intracorporeal triangulation in 6 mm fully wristed, elbowed instruments. The DaVinci RSP system offers enhanced maneuverability, resembling that of multi-port surgery, which facilitates more precise surgical procedures.

Learning curves help in understanding how surgical proficiency develops over time. They provide insights into the number of procedures required for a surgeon to achieve competence and mastery in a particular surgical technique. This is vital for ensuring patient safety and optimizing surgical outcomes. By analyzing learning curves, we can correlate the surgeon’s experience to patient outcomes. Additionally, hospitals and training institutions can use learning curve data to allocate resources more effectively. Understanding the time and case volume required to achieve proficiency can help in planning the necessary infrastructure and support systems to train surgeons efficiently. Therefore, this study aims to analyze the learning curves and associated surgical outcomes of ovarian cystectomy performed using the LESS, RSS, and RSP techniques by a single surgeon.

## 2. Materials and Methods

### 2.1. Study Population

**Figure 1:**
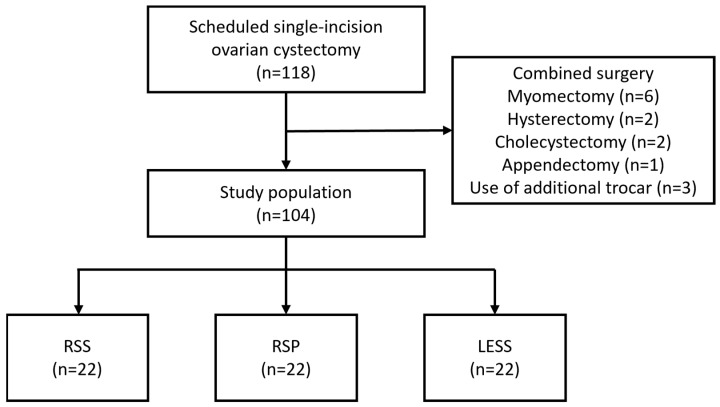
Eligibility of the study population. After excluding 14 ineligible patients, 104 patients were enrolled in the study. LESS; laparo-endoscopic single-site surgery, RSP; Robotic Single-Port system, RSS; Robotic Single-Site platform.

This retrospective study reviewed 118 patients with ovarian tumors who underwent ovarian cystectomy using either LESS, RSS, or RSP at our institution (Figure 1). Fourteen patients were excluded due to the use of an additional port or combined surgery. Ultimately, from January 2021 to December 2023, 22 patients underwent RSP, 52 underwent RSS, and 30 underwent LESS, all performed by a single surgeon. The characteristics and performance metrics evaluated include age, BMI, tumor size, hemoglobin drop, operative time, docking time, console time, and the side of the tumor.

### 2.2. Surgical Techniques

In the LESS procedure, a 15–20 mm umbilical incision was created using the Hasson method. The Lapsingle trocar (Sejong Medical, Paju, Republic of Korea) was employed for instrument insertion. Ovarian cystectomy was defined as a procedure where only cystic tumor lesions were peeled away, preserving all intact ovarian tissue. The tumors were excised through cystectomy, maintaining the residual ovary. Delayed absorbable suture devices (Monofix^®^, manufactured by Samyang Biopharmaceuticals Corporation, Gongju, Republic of Korea) were utilized in all patients for remnant ovarian sutures, performed using the intra-corporeal continuous running suture technique. After cystectomy, the shape of the residual ovary could vary, so the surgeon made an effort to apply a consistent suturing method to minimize surgical technique variations. We started suturing from the side closest to the utero-ovarian ligament and proceeded towards the infundibulopelvic ligament. If bleeding persisted after suturing, additional compression sutures were applied outside the capsule of the sutured ovary to control the bleeding. All resected tumors were removed using a surgical bag to avoid tissue dissemination. After surgery, the umbilical incision was closed layer-by-layer from fascia to skin.

A DaVinci Si or Xi surgical system (Intuitive Surgical Inc., Sunnyvale, CA, USA) was used for RSS and a DaVinci SP system was used for RSP. This included the same surgical procedures as LESS, but one of the single-incision entry systems, such as Lapsingle Vision SP (SEJONG Medical Co., Paju, Republic of Korea), UNI-PORT SP (DALIM Medical Co., Bucheon, Republic of Korea), or Glove Port SP (NELIS Co., Bucheon, Republic of Korea), was used for instrument application. The surgical procedures were consistent with those used in LESS. All resected tumors were also removed using a surgical bag. At the conclusion of the surgery, the umbilical incision was closed using the same method.

### 2.3. Definitions of Terms

For the analysis of the operative times, several intervals were defined. Docking time (DT) was defined as the time taken to dock the robot to the patient. Console time (CT) encompassed the time taken by the surgeon sitting at the console to perform tumor resection and intracorporeal suturing. The total operation time (OT) was defined as the time from incision to closure. Analysis was performed based on these defined intervals.

### 2.4. Statistical Analysis

Variables such as patients’ age, body mass index (BMI), histological test results, operative time, estimated blood loss, days of hospitalization, and complications were analyzed with the ethical board’s approval. Learning curves were analyzed across consecutive cases using the cumulative sum (CUSUM) method. CUSUM analysis was used to quantitatively assess the learning curves of the docking time, console time, and operation time (CUSUM-DT, CUSUM-CT, and CUSUM-OT, respectively). This technique provides graphical information on the trend in the outcome of consecutive procedures performed over time, as it is a plot of the cumulative total of differences between each data point and the mean of all data points. This approach gives a visual representation of the learning curve.

We used the Statistical Package for the Social Sciences 25.0 (SPSS Inc., Chicago, IL, USA) for statistical analysis. The Kolmogorov–Smirnov test was used to verify standard normal distributional assumptions. The Student’s *t*-test and Mann–Whitney U test were used for the parametric and non-parametric variables, respectively. Differences between proportions were compared using a Fisher’s exact test or χ^2^ test. *p* < 0.05 was considered statistically significant.

## 3. Results

### 3.1. Patient Demographics and Tumor Characteristics

Table 1 shows the patients’ demographics and characteristics. The average age across the groups did not show significant variation and the average body mass index was similar across all groups. The average tumor size was significantly larger in the RSS group compared to RSP and LESS. There was a notable difference in the side of the tumor (*p* = 0.006), with the LESS group exhibiting a higher rate of occurrence on the right side (*p* = 0.011).

### 3.2. Surgical Outcomes

The surgical outcomes of the study population are shown in Table 2. The transfusion rate and level of hemoglobin drop showed no significant differences among the groups. The length of stay was also not different between groups. In terms of total operative time, LESS demonstrated the shortest operative time, while RSS and RSP exhibited comparable times (RSS: 99.13 min, RSP: 111.59 min, LESS: 72.50 min; *p* = 0.004). The average docking time showed no significant difference between RSS and RSP (RSS: 3.77 min, RSP: 3.09 min; *p* = 0.245). Finally, the console time was slightly longer for RSP compared to RSS; however, it showed no significant difference (RSS: 52.83 min, RSP: 63.72 min; *p* = 0.271).

### 3.3. Learning Curve Analysis

#### 3.3.1. Docking Time Learning Curve

**Figure 2:**
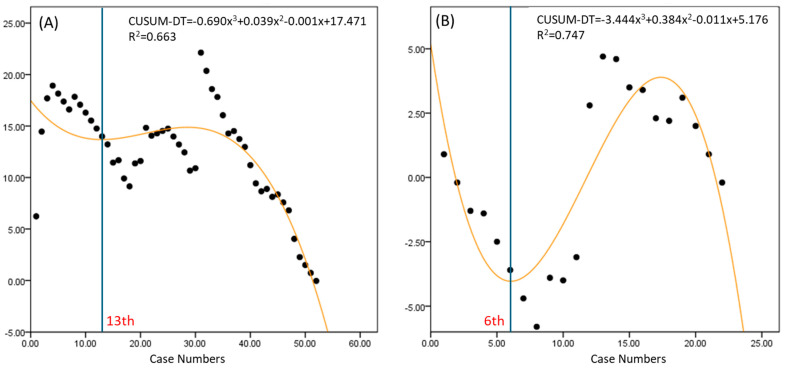
Learning curves for docking time in robotic surgery. (**A**) Robotic Single-Site platform, (**B**) Robotic Single-Port system. CUSUM-DT; cumulative sum of docking time.

RSS exhibited a wide range of docking times, indicating variability in difficulty (Figure 2). The learning curve was achieved by the 13th case for RSS and the 6th case for RSP, suggesting a faster learning curve for RSP.

#### 3.3.2. Console Time Learning Curve

**Figure 3:**
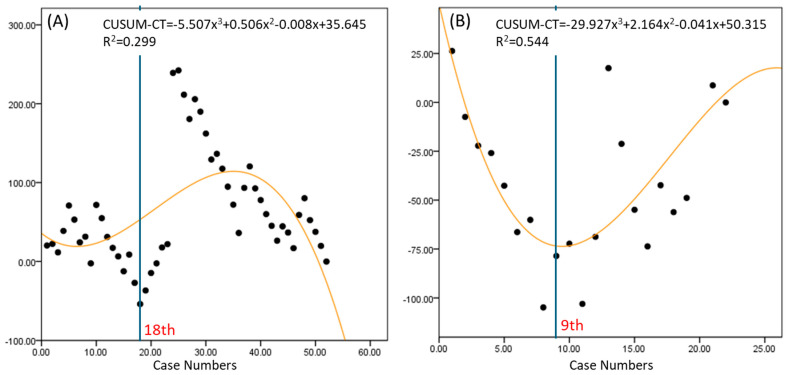
Learning curves for console time in robotic surgery. (**A**) Robotic Single-Site platform, (**B**) Robotic Single-Port system. CUSUM-CT; cumulative sum of console time.

The console time for the RSS group reached proficiency at the 18th case, while the RSP group reached proficiency at the 9th case, indicating that the RSP group achieved proficiency more quickly (Figure 3). Additionally, the variation in the curve is greater for the RSS group, suggesting that the console time fluctuates more with each case in the RSS group. This greater variability is likely to be a factor contributing to the slower improvement in proficiency seen for the RSS group.

#### 3.3.3. Operation Time Learning Curve

**Figure 4:**
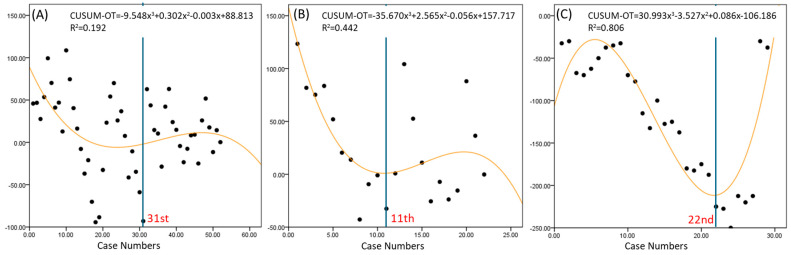
Learning curves for operation time in robotic surgery. (**A**) Robotic Single-Site platform, (**B**) Robotic Single-Port system, (**C**) laparo-endoscopic single-site surgery. CUSUM-OT; cumulative sum of operation time.

The learning curves for operative time showed that RSP achieved proficiency by the 11th case, followed by LESS at the 22nd case, and RSS at the 31st case (Figure 4). LESS exhibited the greatest variability in operative time, followed by RSS and then RSP.

## 4. Discussion

Only a few studies exist comparing conventional single-site laparoscopy to robotic single-incision surgery. Lee et al. investigates the effectiveness of RSS ovarian cystectomy compared to LESS surgery. The study analyzed preoperative and postoperative anti-Müllerian hormone (AMH) levels to assess ovarian reserves. The results showed that the decrease in AMH levels was significantly lower in the RSS group, suggesting better fertility preservation. Additionally, RSS proved feasible without additional ports, even in more complex cases, highlighting its potential advantages in preserving ovarian function in complex surgeries [9]. Another study compared RSS surgery and LESS surgery for ovarian cystectomy, which found no significant differences in bleeding, postoperative length of stay, or complications between the two methods [10]. However, RSS had a longer surgical time.

To our knowledge, this study is the first to compare the learning curves of LESS, RSS, and RSP for ovarian cystectomy simultaneously. The surgeon for the patients included in this study has been performing multiport laparoscopic surgeries for some time. However, single-port laparoscopic and single-port robotic surgeries were initiated within the last five years. The surgeon has primarily performed hysterectomies and myomectomies and began applying these techniques to adnexal surgeries during the period covered by this study.

By examining patient demographics, tumor characteristics, and detailed performance metrics, this research provides a comprehensive overview of the strengths and limitations of each surgical approach, particularly focusing on the proficiency learning curve. This study found no significant differences in age, BMI, or parity among the groups, indicating that the patient demographics were well matched. However, there were significant differences in tumor size and location. The RSS group had larger tumors on average, which might have contributed to the longer operative times observed in this group. The higher incidence of right-sided tumors in the LESS group might suggest a predisposition for selecting LESS for such cases, possibly due to anatomical or ergonomic reasons.

The operative times varied significantly across the techniques, with LESS demonstrating the shortest time, followed by RSS and RSP. LESS’s shorter operative time might be attributed to its relatively simpler setup and fewer instruments compared to the robotic platforms. However, the robotic systems (RSS and RSP) showed advantages in terms of precision and maneuverability, which are crucial in complex cases requiring delicate tissue handling and suturing. Despite the longer operative times, the robotic systems did not significantly differ from LESS in terms of transfusion rates, hemoglobin drop, or length of hospital stay, suggesting that the increased operative time did not adversely impact immediate postoperative recovery. This is consistent with previous studies that highlighted the benefits of robotic systems in reducing surgeon fatigue and improving ergonomics, which could translate into better patient outcomes over time [9,11,12,13].

Several studies have compared LESS to RSS in the field of gynecology. A recent study compared the surgical outcomes of single-site hysterectomy for benign conditions using either the LESS or RSS technique [14]. The study found that while LESS had shorter operative times, RSS showed advantages in terms of reduced intraoperative blood loss and shorter hospital stays. There were no significant differences in adverse events between the two groups, indicating that both approaches are safe and effective, but that RSS may offer better outcomes in specific scenarios. A review and meta-analysis evaluated the feasibility and outcomes of robotic single-site hysterectomy (RSSH) compared to laparo-endoscopic single-site hysterectomy (LESSH) [15]. The findings suggested that RSSH might provide improved visualization and ergonomics, potentially leading to better surgical precision and outcomes. However, it was noted that operative times for RSSH were generally longer than for LESSH, though the differences in clinical outcomes were minimal. Another recent study compared the outcomes of LESS and RSP for procedures including hysterectomy, ovarian cystectomy, and myomectomy [3]. The findings indicated that RSP had comparable surgical outcomes to LESS. While RSP showed shorter operation times and fewer postoperative hemoglobin changes, the differences were not statistically significant. The study concluded that RSP is a feasible and safe option for gynecologic surgeries. Additionally, there is a study that specifically examined the performance of RSS versus LESS in performing complex tasks such as suturing and knot-tying [16]. The findings highlighted that RSS provided better ergonomics and precision, reducing the technical difficulty of such tasks. This suggests that for procedures requiring intricate suturing, RSS might be more advantageous than LESS. These articles provide reasonable evidence that robotic single-incision surgeries could have benefits in the field of minimally invasive gynecologic surgery.

The learning curve analysis using the CUSUM method provided valuable insights into the proficiency acquisition for each technique [17]. The RSP system showed the fastest learning curve for docking time, console time, and overall operative time, achieving proficiency by the 6th, 9th, and 11th cases, respectively. This rapid learning curve can be attributed to the advanced design of the RSP system, which offers enhanced maneuverability and more intuitive control through its EndoWrist SP instruments. In contrast, the RSS system required more cases to reach proficiency (13th case for docking time, 18th for console time, and 31st for overall operative time). The variability in the RSS learning curves suggests that surgeons experienced greater fluctuations in performance, possibly due to the system’s limitations in instrument articulation and triangulation compared to RSP. In comparison to RSS, the RSP shows a steep negative curve in console time from the beginning, indicating that the surgery speed improves more easily in typical cases. The RSS system, while still advantageous over traditional laparoscopic techniques, shows that technological limitations can significantly impact the learning curve. The inability to achieve the same degree of instrument articulation and precision as the RSP system means that surgeons need more time and experience to perform at an optimal level. LESS exhibited the most significant variability and a moderate learning curve, achieving proficiency by the 22nd case. This suggests that while LESS can be efficient once mastered, it requires a longer period for surgeons to overcome its inherent limitations, such as restricted instrument movement and the challenges of intracorporeal suturing through a single port. The range of variability is greatest in LESS, followed by RSS and RSP, respectively. The overall surgery time’s variability across cases is largest in LESS and smallest in RSP. This indicates that, as the complexity of the surgery increases, LESS can take a significantly longer time, while RSS and RSP are more capable of handling complex cases within a reasonable time frame.

The findings from this study have important implications for surgical training and practice. The faster learning curve and better performance metrics associated with the RSP system suggest that investing in more advanced robotic systems can lead to quicker proficiency and potentially better patient outcomes [18]. This is particularly relevant for teaching hospitals and institutions looking to enhance their minimally invasive surgical programs [19]. Moreover, the variability and extended learning curves seen with LESS and RSS highlight the need for targeted training programs and possibly simulation-based practices to help surgeons overcome these challenges more efficiently. Structured training programs that focus on the unique aspects of each technique, combined with mentorship and regular performance evaluations, could accelerate the learning process and improve overall surgical outcomes [20].

While this study provides valuable insights, it has some limitations. This study’s retrospective design may introduce biases related to data collection and interpretation, limiting the ability to establish causality. For example, the differing tumor sizes and locations across the groups could have influenced the results of this study. Although tumor size differences were not intentional and likely did not significantly impact the surgical time or outcomes, the significant prevalence of right ovarian tumors in the LESS group suggests a clear selection bias. In LESS surgery, when operating from the left side of the patient, it is relatively more challenging to operate on the left ovary due to discomfort and increased instrument collisions compared to the right ovary. This likely contributed to the observed results. A prospective study design with an equal distribution of tumor locations could help to address this issue. Furthermore, the findings of this study are based on the experience of a single surgeon, which may not be generalizable to other surgeons with varying levels of expertise and technique preferences. Also, the relatively small sample size may reduce the statistical power of the study, potentially impacting the detection of significant differences between the surgical techniques. Additionally, this study primarily focuses on immediate surgical outcomes, with limited information on long-term follow-ups to assess the impact on patient quality of life, recurrence rates, and overall satisfaction. Finally, this study does not include a comparative cost analysis of each surgical technique. Robotic surgery generally incurs higher costs compared to conventional laparoscopic surgery in most countries. Despite this, many patients in South Korea are enrolled in private insurance plans in addition to the national public insurance. These private plans often cover a significant portion of the costs associated with robotic surgery, reducing the financial burden on patients. Therefore, a cost analysis of each surgical method may not hold substantial clinical significance in the context of the Korean healthcare system. Future research could involve multi-center trials with larger sample sizes and multiple surgeons to validate these results further. Additionally, long-term follow-up studies could assess the impact of these surgical techniques on patient quality of life, recurrence rates, and overall satisfaction.

## 5. Conclusions

In conclusion, this study underscores the significant advancements in robotic surgery and their impact on learning curves and surgical outcomes. The RSP system’s superior design facilitates faster proficiency and more consistent performance, suggesting that it is a preferable choice for complex minimally invasive procedures like ovarian cystectomy. However, the choice of surgical technique should consider individual patient characteristics, surgeon experience, and available resources to optimize outcomes.

## Figures and Tables

**Table 1:** Patient demographics and characteristics.

Variable	RSS (*n* = 52)	RSP (*n* = 22)	LESS (*n* = 30)	*p*-Value
Age (years)	28.87 ± 6.91	28.32 ± 7.74	32.07 ± 11.48	0.196
BMI (kg/m^2^)	22.67 ± 4.57	21.15 ± 3.53	21.67 ± 3.21	0.273
Parity				0.579
Virgin	8 (15.38)	4 (18.18)	3 (10.00)	
Nulliparous	33 (63.46)	16 (72.73)	19 (63.33)	
Multiparous	11 (21.15)	2 (9.09)	8 (26.67)	
History of abdominal surgery				0.637
Present	4 (7.69)	3 (13.64)	2 (6.67)	
Absent	48 (92.31)	19 (86.36)	28 (93.33)	
Tumor size on imaging (cm)	6.5 ± 2.99	4.8 ± 1.61	4.96 ± 2.12	0.006
Tumor location				0.011
Right	25 (48.08)	8 (36.36)	23 (76.67)	
Left	15 (28.85)	5 (22.73)	3 (10.00)	
Bilateral	12 (23.08)	9 (40.91)	4 (13.33)	
Histology				0.085
Teratoma	24 (46.15)	6 (27.27)	12 (40.00)	
Endometrioma	17 (32.69)	12 (54.55)	6 (20.00)	
Cystadenoma	8 (15.38)	2 (9.09)	8 (26.67)	
Borderline tumor	0 (0.00)	1 (4.55)	0 (0.00)	
Miscellaneous *	3 (5.77)	1 (4.55)	4 (13.33)	

Variables were shown as mean±standard deviation or *n* (%). BMI; body-mass index, LESS; laparo-endoscopic single-site surgery, RSP; Robotic Single-Port system, RSS; Robotic Single-Site platform. * Miscellaneous histology includes fibroma and hemorrhagic corpus luteum.

**Table 2:** Surgical outcomes of single-incision ovarian cystectomy.

Variable	RSS (*n* = 52)	RSP (*n* = 22)	LESS (*n* = 30)	*p*-Value
Transfusion, yes	2 (3.85)	0 (0.00)	1 (3.33)	0.655
Hemoglobin-drop (g/dL)	1.72 ± 0.91	1.73 ± 1.06	1.62 ± 0.83	0.875
Length of postoperative stay (days)	2.54 ± 0.96	2.50 ± 0.96	2.26 ± 0.78	0.418
Operation time (minutes)	99.13 ± 40.51	111.59 ± 52.29	72.50 ± 40.78	0.004
Docking time (minutes)	3.77 ± 2.49	3.09 ± 1.66	N/A	0.245
Console time (minutes)	52.83 ± 39.76	63.72 ± 35.65	N/A	0.271
Umbilical hernia, yes	1 (1.92)	0 (0.00)	0 (0.00)	0.604
Wound problem, yes	2 (3.85)	0 (0.00)	1 (3.33)	0.655
Febrile event (≥37.5 °C)	16 (30.77)	5 (22.73)	9 (30.00)	0.773

Variables were shown as mean ± standard deviation or *n* (%). LESS; laparo-endoscopic single-site surgery, N/A; not available, RSP; Robotic Single-Port system, RSS; Robotic Single-Site platform.

## Data Availability

The data presented in this study are available upon request from the corresponding author.

## References

[1] Alammari R., Lightfoot M., Hur H.-C. (2017). Impact of Cystectomy on Ovarian Reserve: Review of the Literature. J. Minim. Invasive Gynecol..

[2] Moon H.-S., Shim J.E., Lee S.R., Jeong K. (2018). The Comparison of Robotic Single-Site Surgery to Single-Port Laparoendoscopic Surgery for the Treatment of Advanced-Stage Endometriosis. J. Laparoendosc. Adv. Surg. Tech..

[3] Kim J.-M., Lee S.-M., Seol A., Song J.-Y., Ryu K.-J., Lee S., Park H.-T., Cho H.-W., Min K.-J., Hong J.-H. (2023). Comparison of Surgical Outcomes between Single-Port Laparoscopic Surgery and Da Vinci Single-Port Robotic Surgery. J. Pers. Med..

[4] Ranjan A., Joshi K.S., Pajai S., Mohammad S. (2022). Laparoendoscopic Single-Site Surgery (LESS): A Shift in Gynecological Minimally Invasive Surgery. Cureus.

[5] Liu Y., Xiong J., Chen Y., Yi Y., Zhang W. (2022). Advances in the Application of Robotic single-site Laparoscopy in Gynecology. Intell. Surg..

[6] Kim S., Min K.J., Lee S., Hong J.H., Song J.Y., Lee J.K., Lee N.W. (2021). Learning Curve could Affect Oncologic Outcome of Minimally Invasive Radical Hysterectomy for Cervical Cancer. Asian J. Surg..

[7] Yoo H.K., Cho A., Cho E.H., Kim S.J., Shim J.E., Lee S.R., Jeong K., Moon H. (2020). Robotic Single-site Surgery in Benign Gynecologic Diseases: Experiences and Learning Curve Based on 626 Robotic Cases at a Single Institute. J. Obstet. Gynaecol. Res..

[8] Arcieri M., Romeo P., Vizzielli G., Restaino S., Driul L., Stabile G., Granese R., Cianci S., Ercoli A. (2023). Robotic Single-Port da Vinci Surgical System (SP1098) in Gynecologic Surgery: A Systematic Review of Literature. Clin. Exp. Obstet. Gynecol..

[9] Lee J.H., Park S.Y., Jeong K., Yun H.Y., Chung H.W. (2023). What is the Role of Robotic Surgery in Ovarian Cystectomy with Fertility Preservation?. J. Robot. Surg..

[10] Kim S., Min K.J., Lee S., Hong J.H., Song J.Y., Lee J.K., Lee N.W. (2020). Robotic single-site Surgery versus Laparo-endoscopic Single-site Surgery in Ovarian Cystectomy: A Retrospective Analysis in Single Institution. Gynecol. Robot. Surg..

[11] Chatterjee S., Das S., Ganguly K., Mandal D. (2024). Advancements in Robotic Surgery: Innovations, Challenges and Future Prospects. J. Robot. Surg..

[12] Yeung T.M., Larkins K.M., Warrier S.K., Heriot A.G. (2024). The Rise of Robotic Colorectal Surgery: Better for Patients and Better for Surgeons. J. Robot. Surg..

[13] Wong S.W., Ang Z.H., Yang P.F., Crowe P. (2021). Robotic Colorectal Surgery and Ergonomics. J. Robot. Surg..

[14] Gardella B., Dominoni M., Gritti A., Mereu L., Bogliolo S., Torella M., Fanfani F., Malzoni M., Couso A., Zapico A. (2023). Comparison between Robotic Single-Site and Laparoendoscopic Single-Site Hysterectomy: Multicentric Analysis of Surgical Outcomes. Medicina.

[15] Prodromidou A., Spartalis E., Tsourouflis G., Dimitroulis D., Nikiteas N. (2020). Robotic Versus Laparoendoscopic Single-site Hysterectomy: A Systematic Review and Meta-analysis. J. Robot. Surg..

[16] Eisenberg D., Vidovszky T.J., Lau J., Guiroy B., Rivas H. (2013). Comparison of Robotic and Laparoendoscopic Single-site Surgery Systems in a Suturing and Knot Tying Task. Surg. Endosc..

[17] Stern N., Li Y., Wang P.Z., Dave S. (2022). A Cumulative Sum (CUSUM) Analysis Studying Operative Times and Complications for a Surgeon Transitioning from Laparoscopic to Robot-assisted Pediatric Pyeloplasty: Defining Proficiency and Competency. J. Pediatr. Urol..

[18] Talamini S., Halgrimson W.R., Dobbs R.W., Morana C., Crivellaro S. (2020). Single Port Robotic Radical Prostatectomy versus Multi-port Robotic Radical Prostatectomy: A Human Factor Analysis During the Initial Learning Curve. Int. J. Med Robot. Comput. Assist. Surg..

[19] Green C.A., Levy J.S., Martino M.A., Porterfield J.R. (2020). The Current State of Surgeon Credentialing in the Robotic Era. Ann. Laparosc. Endosc. Surg..

[20] Walker R.J.B., Stukel T.A., de Mestral C., Nathens A., Breau R.H., Hanna W.C., Hopkins L., Schlachta C.M., Jackson T.D., Shayegan B. (2023). Hospital Learning Curves for Robot-assisted Surgeries: A Population-based Analysis. Surg. Endosc..

